# On topological analysis of two-dimensional covalent organic frameworks via M-polynomial

**DOI:** 10.1038/s41598-024-57291-9

**Published:** 2024-03-23

**Authors:** Hong Yang, Muhammad Farhan Hanif, Muhammad Kamran Siddiqui, Mazhar Hussain, Nazir Hussain, Samuel Asefa Fufa

**Affiliations:** 1 School of Computer Science, Chengdu University, Chengdu, China; 2 Department of Mathematics and Statistics, The University of Lahore, Lahore Campus, Lahore, Pakistan; 3 Department of Mathematics, COMSATS University Islamabad, Lahore Campus, Lahore, Pakistan; 4 Department of Mathematics, Addis Ababa University, Addis Ababa, Ethiopia

**Keywords:** Degree-based indices, ZnP-COF, Degree of vertex, M-polynomial, Applied mathematics, Physical chemistry, Applied mathematics, Physical chemistry

## Abstract

Covalent organic frameworks (ZnP-COFs) made of zinc-porphyrin have become effective materials with a variety of uses, including gas storage and catalysis. To simulate the structural and electrical features of ZnP-COFs, this study goes into the computation of polynomials utilizing degree-based indices. We gave a methodical study of these polynomial computations using Excel, illustrating the complex interrelationships between the various indices. Degree-based indices provide valuable insights into the connectivity of vertices within a network. M-polynomials, on the other hand, offer a mathematical framework for representing and studying the properties of 2D COFs. By encoding structural information into a polynomial form, M-polynomials facilitate the calculation of various topological indices, including the Wiener index, Zagreb indices, and more. The different behavior of ZnP-COFs based on degree-based indices was illustrated graphically, and this comparison provided insightful information for prospective applications and the construction of innovative ZnP-COF structures. Moreover, we discuss the relevance of these techniques in the broader context of materials science and the design of functional covalent organic frameworks.

## Introduction

The study of relationships between items represented as vertices and edges and the connections between these objects is the subject of graph *G*. The degree of vertices *u*, which describes the number of edges incident to a vertex and is denoted by $d_{u}$, is one of the fundamental ideas in graph theory^1^. Understanding a graph’s connectivity and structural characteristics depends on the degree of its vertices. It offers perceptions of how a network’s resilience and behavior are affected by the complexity of its relationships. A graph size, or the total number of vertices and edges it contains, is another crucial feature. The number of vertices in the graph is also indicated by the sequence in which it is constructed. The understanding of graph topologies is improved by all of these ideas, which also aid with a number of applications, including social network modeling, chemical structure analysis, and transportation system optimization^2^.

Using ideas from graph theory, the interdisciplinary area of chemical graph theory analyses and explains the structures and behaviors of molecules. “A molecular graph consists of atoms considered as vertices and bonds of atoms considered as edges^3^. This approach permits the systematic investigation of molecular properties using topological indices, which provide quantifiable measurements of size, symmetry, and complexity. For detecting isomers, simulating chemical reactions, and projecting properties, chemical graph theory is crucial. It encompasses numerous chemicals, materials, and molecular networks, advancing our comprehension of molecular behavior and assisting in applications like medicine development and the creation of new materials. Modern computational chemistry relies heavily on graph-based models because they make it easier to explore, create, and optimize molecules for a variety of uses^4^.

**Figure 1:**
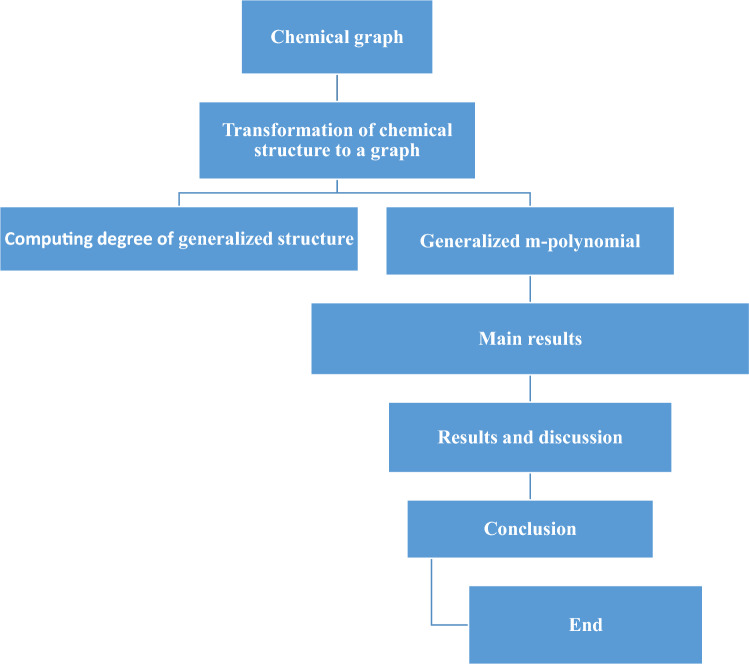
Flowchart to measure a m-polynomial.

The methodology’s flowchart is displayed in Fig. 1. Topological indices are numbers obtained from graph topologies and provide information on the connectivity and characteristics of systems, networks, and compounds^5^. They have numerous uses in a variety of disciplines, such as chemistry, biology, and computer science. The sum of the shortest paths in a graph is measured by the Wiener index, which reflects the complexity and size of molecules. Based on nearby vertex degrees and bond multiplicities, the Randi ć index makes predictions about characteristics. By capturing vertex degrees and degree products, Zagreb indices can reveal symmetry and complexity. These indices offer quantitative details about buildings, assisting with compound design and property prediction^6^. Technological advancements make the ability to apply computing to complex systems possible, underscoring its expanding significance in cross-disciplinary studies^7^.

**Figure 2:**
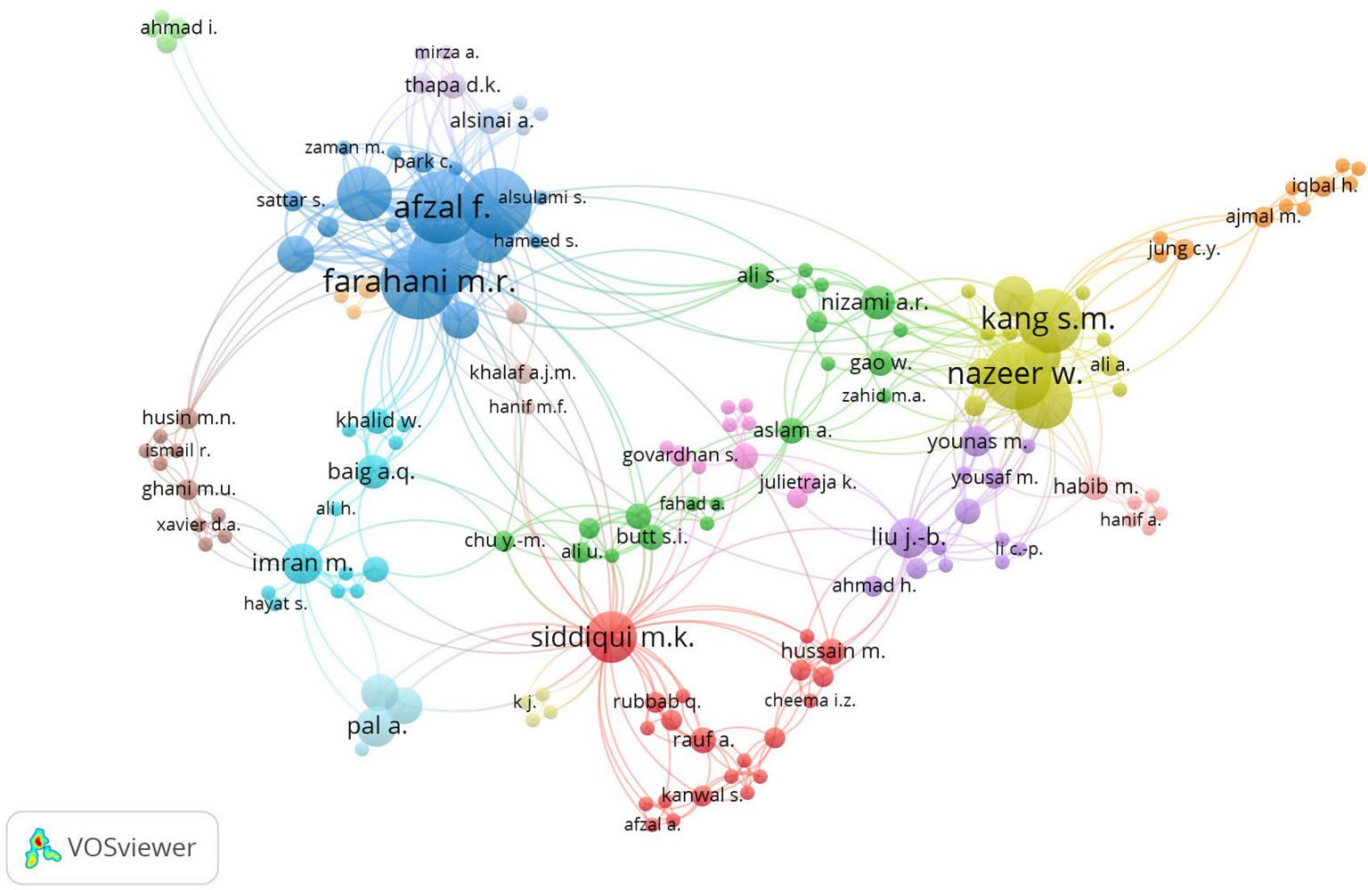
Bibilography analysis for an author working on M-polynomial.

A map with several authors each working on m polynomials (based on the Scopus database https://www.scopus.com/) connected is depicted in Fig. 2. The collaborative network between authors working on m polynomials is depicted in this picture. The thickness of the edge denotes the number of co-publications between the two authors, while the size of the node denotes the author’s total number of publications. The image demonstrates the many author groupings working on m polynomials. Farhani M.R., who has co-authored works with many of the other authors in the figure, is the focal point of one cluster. Mirza A., who has written papers with many of the other authors, is the center of another cluster. A group of writers is also concentrated around Thapa, D.K.

**Figure 3:**
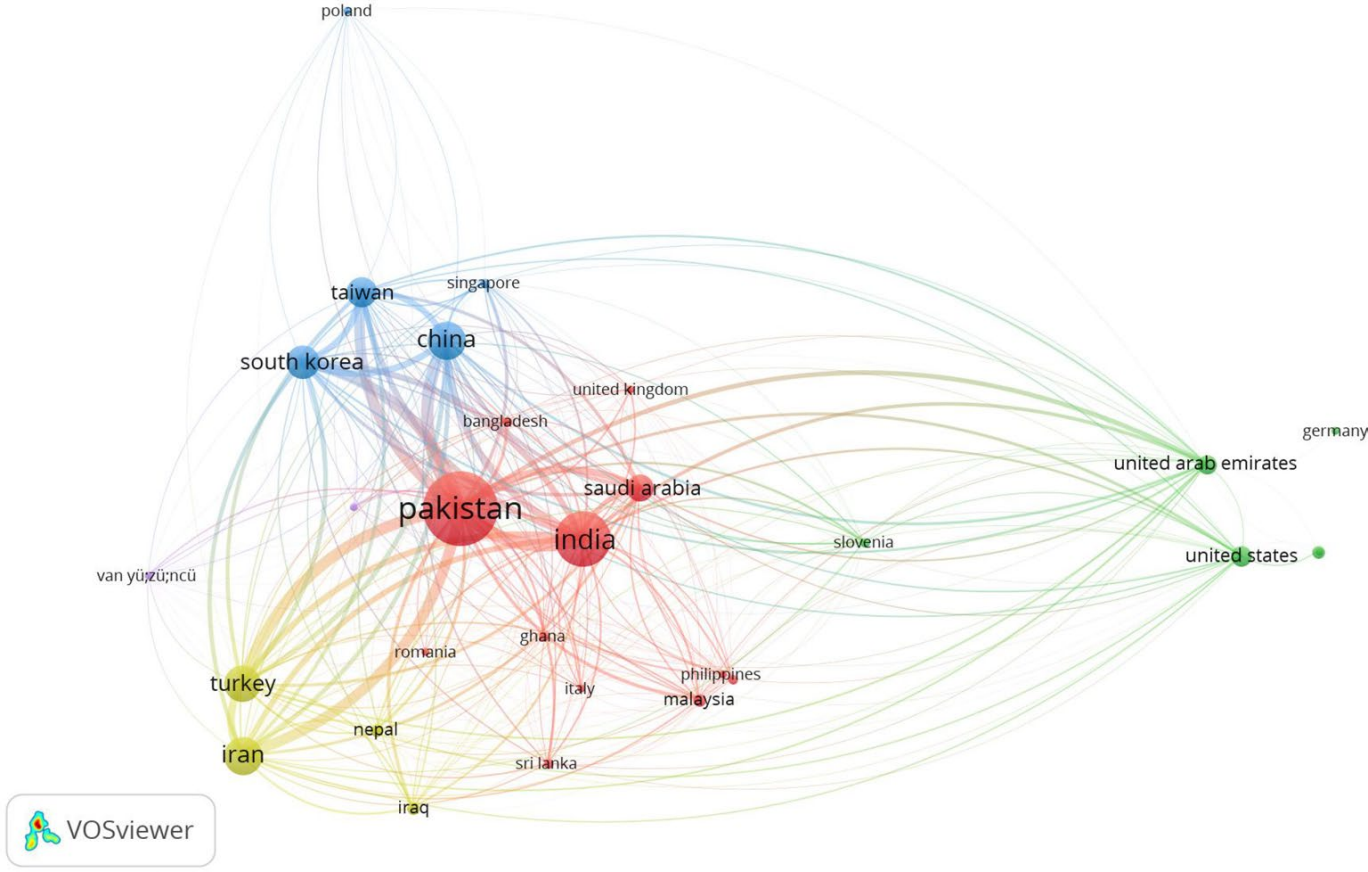
Bibilography analysis for an author working on m polynomial.

The partnerships between scholars from those nations on m-polynomials are represented by the lines separating the countries. The number of partnerships between the two countries is shown by the line’s thickness. Figure 3 demonstrates that Pakistan is the nation that is most engaged in m-polynomial research. Many of the other nations shown in the picture, such as China, South Korea, India, and the United Kingdom, are partners with it. India is a significant contributor to the study of m-polynomials. It collaborates with a large number of the other nations shown in the graph, including the US, South Korea, Japan, and China.

Afzal et al.^8^ computed the M-polynomial using the degree-based indices. Raza et al.^9^ discussed the degree-based polynomial of some nanostructures. Jahangeer Baig et al.^10^ analyzed the important class of graphs using degree-based indices and polynomials. Koam and Ahmad discussed the polynomials for three-dimensional mesh networks. Hasan A et al.^11^ for the X-level wheel graph, the polynomial based on proximity and angle was determined. Julietraja et al.^12^ used m-polynomial analysis to carry out the computation of topological descriptors for coronoid systems. Ghani et al.^13^ used valency-based m-polynomial analysis to investigate the concise representation of pharmaceuticals. Sarkar et al.^14,15^ discussed the m-polynomial.

Rasool et al.^16^ related $ve-$ indices and $M_ {ve} -$Polynomial of r- Regular Simple Graph. Xavier et al.^17^ used the neighborhood M-Polynomial technique to have a conversation about the chemical descriptors of penta heptagonal nanostructures. Balasubramanian has established the relationship between entropies, topological indices, graph spectra, Laplacians, and matching polynomials in the context of n-dimensional hypercubes. Chu et al.^18^ calculated the benzenoid triangular and hourglass system’s Zagreb type polynomial. Hakami et al.^19^ M-polynomials were used as a crucial analytical tool to conduct an extensive investigation of two-dimensional coronene fractal structures. Different M-polynomial and topological descriptors are defined in Table 1.

**Table 1:** Degree-based indices and their polynomials.

Topological indices	$f(\tilde{a},\tilde{b})$	$M(G,\tilde{a},\tilde{b})$
First Zagreb index	$\tilde{a}+\tilde{b}$	$M_1(G,\tilde{a},\tilde{b})$=$(D_{\tilde{a}}+D_{\tilde{b}})M(G,\tilde{a},\tilde{b})|_{\tilde{a}=\tilde{b}=1}$
Second Zagreb index	$\tilde{a}\tilde{b}$	$M_2(G,\tilde{a},\tilde{b})=(D_{\tilde{a}}D_{\tilde{b}})M(G,\tilde{a},\tilde{b})|_{\tilde{a}=\tilde{b}=1}$
Second modified Zagreb index	$\frac{1}{{\tilde{a}}{\tilde{b}}}$	$^mM_2(G,\tilde{a},\tilde{b})=(\delta _{\tilde{a}}\delta _{\tilde{b}})M(G,\tilde{a},\tilde{b})|_{\tilde{a}=\tilde{b}=1}$
General Randić index, ${\tilde{a}}\ne 0$	$({\tilde{a}\tilde{b}})^{\alpha }$	$R_{\alpha }(G)=(D^{\alpha }_{\tilde{a}}D^{\alpha }_{\tilde{b}})M(G,\tilde{a},\tilde{b})|_{\tilde{a}=\tilde{b}=1}$
Inverse general Randić index, ${\tilde{a}}\ne {0}$	$\frac{1}{({\tilde{a}}{\tilde{b}})^{\alpha }}$	$RR_{\alpha }(G,\tilde{a},\tilde{b})=(\delta {\tilde{a}}^{\alpha }_{\tilde{a}}\delta {\tilde{a}}^{\alpha }_{\tilde{b}})M(G,\tilde{a},\tilde{b})|_{\tilde{a}=\tilde{b}=1}$
Symmetric division index	$\frac{{\tilde{a}}^2+{\tilde{b}}^2}{{\tilde{a}}{\tilde{b}}}$	$SS D(G)=|D_{\tilde{a}}\delta _{\tilde{b}}+D_{\tilde{b}}\delta _{\tilde{a}}|_{\tilde{a}=\tilde{b}=1}$
Harmonic index	$\frac{2}{{\tilde{a}}+{\tilde{b}}}$	$H(G)=2\delta _{\tilde{b}}JM(G,\tilde{a},\tilde{b})|_{{\tilde{a}}=1}$
Inverse sum index	$\frac{{\tilde{a}}{\tilde{b}}}{{\tilde{a}}+{\tilde{b}}}$	$I(G)=\delta _{\tilde{a}} JD_{\tilde{a}}D_{\tilde{b}}M(G,\tilde{a},\tilde{b})|_{{\tilde{a}}=1}$

$$D_{\tilde{a}}={\tilde{a}}(\delta /\delta _{\tilde{a}})M(G,{\tilde{a}},{\tilde{b}})|_{\tilde{a}=\tilde{b}=1}$$,$$D_{\tilde{b}}={\tilde{b}}(\delta /\delta _{\tilde{b}})M(G,{\tilde{a}},{\tilde{b}})|_{\tilde{a}=\tilde{b}=1}$$,$$\delta _{\tilde{a}}=\int _{0}^{{\tilde{a}}}M(G,x,{\tilde{b}})/x dx$$,

$$\delta _{\tilde{b}}=\int _{0}^{{\tilde{b}}}M(G,{\tilde{a}},y)/y dy$$,$$J=(M;{\tilde{a}},{\tilde{a}})$$,$$Q_{\tilde{a}}=x^{\tilde{a}}M(G;\tilde{a},\tilde{b}) {\tilde{a}}\ne 0.$$

## Two-dimensional (2D) ZnP-COF structures

A novel family of materials known as porphyrin-based two-dimensional (2D) Znp-COF structures has evolved. These materials combine the special characteristics of porphyrin molecules with the custom architecture of COFs. These structures’ adaptable and diverse qualities make them extremely promising for a wide range of applications. The properties and functionalities of the material can be precisely tuned by adding zinc (Zn) atoms to the COF framework^20^. These porphyrins-based 2D Znp-COFs have outstanding porosity and surface area, which is one of their major characteristics. A porous structure with controlled pore diameters is created by the carefully planned arrangement of organic linkers and porphyrin units, allowing for effective gas adsorption, separation, and storage. Additionally, the porphyrins’ intrinsic-conjugated nature endows the substance with intriguing electrical properties that make it appealing for use in electronics and optoelectronics^21^.

The inclusion of zinc in the COF framework creates more potential paths. Zn can be combined with functional molecules to provide specific amounts of reactivity and selectivity. Due to their adaptable design, Znp-COFs make ideal choices for catalysis, sensing, and drug delivery systems. Furthermore, these hybrid materials are highly suited for addressing present problems in energy conversion and storage due to the functional diversity of porphyrins, stability and structural resilience of COFs, and coordination ability of Zn. The main objective of the research has been to identify the distinguishing structural traits and applications of porphyrin-based 2D Znp-COFs. As we gain a better knowledge of their characteristics, the potential for advancements in fields like sustainable chemistry, photonics, and beyond becomes increasingly evident^22^.

**Figure 4:**
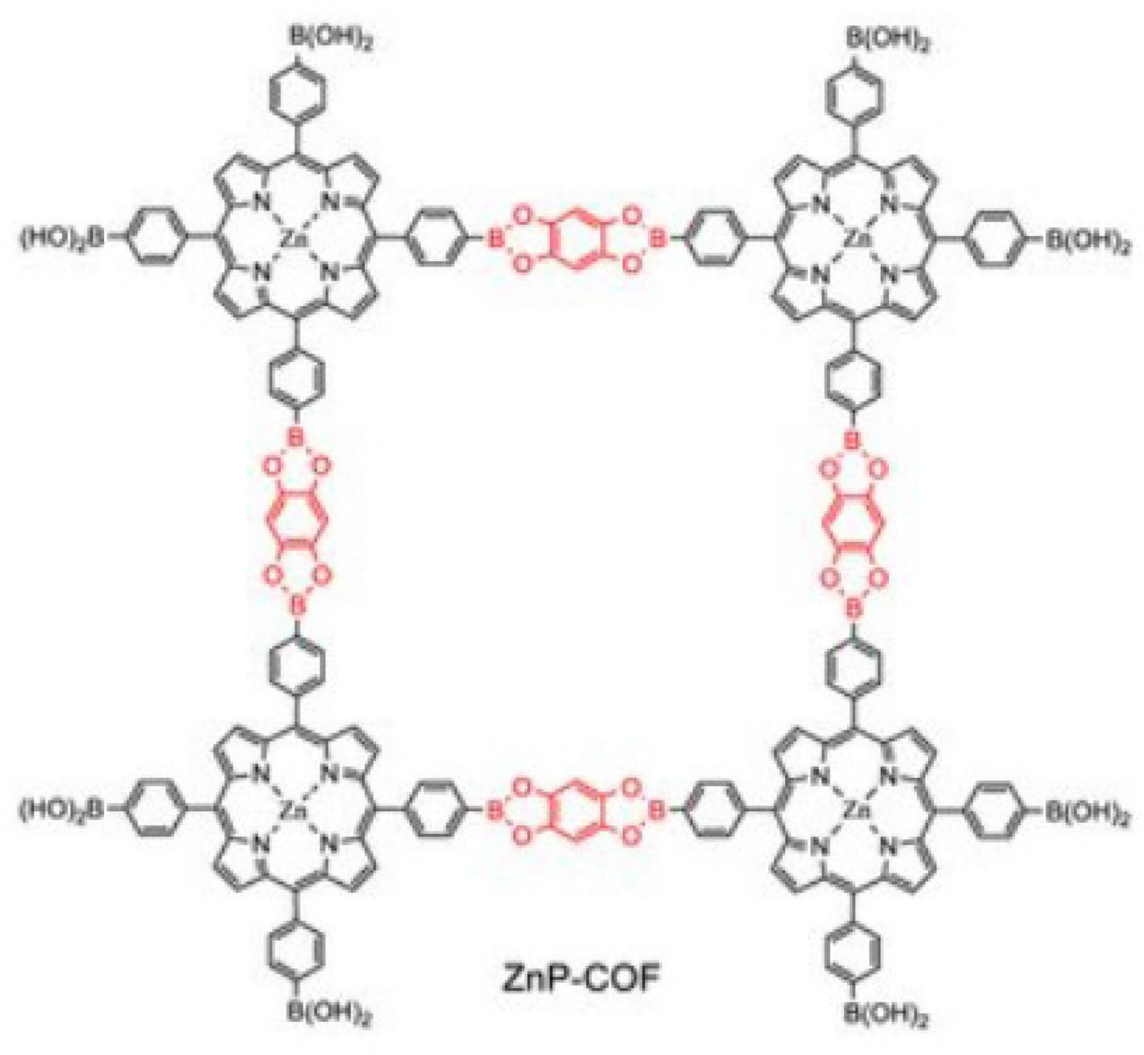
Molecular structure of Porphyrin based two-dimensional covalent organic framework (ZnP-COF)^21^.

These materials serve as an excellent illustration of the extraordinary progress being made at the nexus of organic synthesis, materials science, and nanotechnology, offering up new opportunities for innovation that may completely alter several industrial and technological environments see Fig. 4.

## Main results

To begin this section, we compute a number of degree-based topological indices for the two- dimensional ZnP-COF. Figure 4 illustrates the structure of the two- dimensional ZnP-COF. The method of edge partitioning and vertices degree counting is the main strategy employed here.

### Theorem 1

Crystallographic structure of the graph of $G\approx ZnP-COF[m; n]$, where $m; n\ge 1$

We have:

$$\begin{aligned} M(G,\tilde{a},\tilde{b})= & {} 4(n+m){\tilde{a}\tilde{b}}^{3}+(12mn){\tilde{a}}^{2}{\tilde{b}}^{2}+(64mn-4m-4n){\tilde{a}}^{2}{\tilde{b}}^{3}+(28mn+2m+2n){\tilde{a}}^{3}{\tilde{b}}^{3}+(4mn){\tilde{a}}^{3}{\tilde{b}}^{4} \end{aligned}$$

### Proof

Let *G* be the crystallographic structure of $ZnP{-}COF[m; n]$. The edge set of $ZnP{-}COF$ is given as:

$$\begin{aligned} E_1= & {} E_{(1,3)}=4m+4n\\ E_2= & {} E_{(2,2)}=12mn\\ E_3= & {} E_{(2,3)}=64mn-4m-4n\\ E_4= & {} E_{(3,3)}=28mn+2m+2n\\ E_5= & {} E_{(3,4)}=4mn \end{aligned}$$

Thus, using Table 1, the M-polynomial of $ZnP{-}COF$ is

$$\begin{aligned} M(G,\tilde{a},\tilde{b})= & {} \sum _{1\le 3}m_{13}(G){\tilde{a}}^{1}{\tilde{b}}^{3}+\sum _{2\le 2}m_{22}(G){\tilde{a}}^{2}{\tilde{b}}^{2}+\sum _{2\le 3}m_{23}(G){\tilde{a}}^{2}{\tilde{b}}^{3}+\sum _{3\le 3}m_{33}(G){\tilde{a}}^{3}{\tilde{b}}^{3}+\sum _{3\le 4}m_{34}(G){\tilde{a}}^{3}{\tilde{b}}^{4}\\ M(G,\tilde{a},\tilde{b})= & {} \sum _{pq\in E_1}m_{13}(G){\tilde{a}}^{1}{\tilde{b}}^{3}+\sum _{pq\in E_2}m_{22}(G){\tilde{a}}^{2}{\tilde{b}}^{2}+\sum _{pq\in E_3}m_{23}(G){\tilde{a}}^{2}{\tilde{b}}^{3}+\sum _{pq\in E_4}m_{33}(G){\tilde{a}}^{3}{\tilde{b}}^{3}+\sum _{pq\in E_5}m_{34}(G){\tilde{a}}^{3}{\tilde{b}}^{4}\\ M(G,\tilde{a},\tilde{b})= & {} |E_1(G)|{\tilde{a}}^{1}{\tilde{b}}^{3}+|E_2(G)|{\tilde{a}}^{2}{\tilde{b}}^{2}+|E_3(G)|{\tilde{a}}^{2}{\tilde{b}}^{3}+|E_4(G)|{\tilde{a}}^{3}{\tilde{b}}^{3}+|E_5(G)|{\tilde{a}}^{3}{\tilde{b}}^{4}\\ M(G,\tilde{a},\tilde{b})= & {} 4(n+m){\tilde{a}\tilde{b}}^{3}+(12mn){\tilde{a}}^{2}{\tilde{b}}^{2}+(64mn-4m-4n){\tilde{a}}^{2}{\tilde{b}}^{3}+(28mn+2m+2n){\tilde{a}}^{3}{\tilde{b}}^{3}+(4mn){\tilde{a}}^{3}{\tilde{b}}^{4} \end{aligned}$$

$$\square$$

### Theorem 2

Crystallographic structure of the graph of $G\approx ZnP{-}COF$, where $m; n\ge 1$

We have:

$$\begin{aligned} M_1(G)= & {} 564mn+8(n+m). \end{aligned}$$

### Proof

Suppose that,

$$\begin{aligned} M(G,\tilde{a},\tilde{b})= & {} 4(n+m){\tilde{a}\tilde{b}}^{3}+(12mn){\tilde{a}}^{2}{\tilde{b}}^{2}+(64mn-4m-4n){\tilde{a}}^{2}{\tilde{b}}^{3}+(28mn+2m+2n){\tilde{a}}^{3}{\tilde{b}}^{3}+(4mn){\tilde{a}}^{3}{\tilde{b}}^{4} \end{aligned}$$

is M-Polynomial of $ZnP{-}COF$

$$\begin{aligned} D_{\tilde{a}}=\frac{\partial f}{\partial a}.a \end{aligned}$$

Now

$$\begin{aligned} M(G,\tilde{a},\tilde{b})= & {} 4(n+m){\tilde{a}\tilde{b}}^{3}+(12mn){\tilde{a}}^{2}{\tilde{b}}^{2}+(64mn-4m-4n){\tilde{a}}^{2}{\tilde{b}}^{3}+(28mn+2m+2n){\tilde{a}}^{3}{\tilde{b}}^{3}+(4mn){\tilde{a}}^{3}{\tilde{b}}^{4} \end{aligned}$$

Find $$D_{\tilde{a}}$$

$$\begin{aligned} D_{\tilde{a}}= & {} 4(n+m){\tilde{a}\tilde{b}}^{3}+2(12mn){\tilde{a}}^{2}{\tilde{b}}^{2}+2(64mn-4m-4n){\tilde{a}}^{2}{\tilde{b}}^{3}+3(28mn+2m+2n){\tilde{a}}^{3}{\tilde{b}}^{3}+3(4mn){\tilde{a}}^{3}{\tilde{b}}^{4} \end{aligned}$$

Similarly, find $$D_{\tilde{b}}$$

$$\begin{aligned} D_{\tilde{b}}= & {} 34(n+m){\tilde{a}\tilde{b}}^{3}+2(12mn){\tilde{a}}^{2}{\tilde{b}}^{2}+3(64mn-4m-4n){\tilde{a}}^{2}{\tilde{b}}^{3}+3(28mn+2m+2n){\tilde{a}}^{3}{\tilde{b}}^{3}+4(4mn){\tilde{a}}^{3}{\tilde{b}}^{4} \end{aligned}$$

Using Table 1 we have,

$$\begin{aligned} M_1(G)= & {} [4(n+m)+2(12mn)+2(64mn-4m-4n)+3(28mn+2m+2n)+3(4mn)]\\&\qquad +&[34(n+m)+2(12mn)+3(64mn-4m-4n)+3(28mn+2m+2n)+4(4mn)] \\ M_1(G)= & {} 564mn+8m+8n. \end{aligned}$$

$$\square$$

### Theorem 3

Crystallographic structure of the graph of $G\approx ZnP{-}COF$, where $m; n\ge 1$

We have:

$$\begin{aligned} M_2(G)= & {} 732mn+6m+6n. \end{aligned}$$

### Proof

Suppose that,

$$\begin{aligned} M(G,\tilde{a},\tilde{b})= & {} 4(n+m){\tilde{a}\tilde{b}}^{3}+(12mn){\tilde{a}}^{2}{\tilde{b}}^{2}+(64mn-4m-4n){\tilde{a}}^{2}{\tilde{b}}^{3}+(28mn+2m+2n){\tilde{a}}^{3}{\tilde{b}}^{3}+(4mn){\tilde{a}}^{3}{\tilde{b}}^{4} \end{aligned}$$

is M-Polynomial of $ZnP{-}COF$

Find $$D_{\tilde{a}}$$

$$\begin{aligned} D_{\tilde{a}}= & {} 4(n+m){\tilde{a}\tilde{b}}^{3}+2(12mn){\tilde{a}}^{2}{\tilde{b}}^{2}+2(64mn-4m-4n){\tilde{a}}^{2}{\tilde{b}}^{3}+3(28mn+2m+2n){\tilde{a}}^{3}{\tilde{b}}^{3}+3(4mn){\tilde{a}}^{3}{\tilde{b}}^{4} \end{aligned}$$

Take $$D_{\tilde{b}}$$

$$\begin{aligned} D_{\tilde{b}}D_{\tilde{a}}= & {} 34(n+m){\tilde{a}\tilde{b}}^{3}+4(12mn){\tilde{a}}^{2}{\tilde{b}}^{2}+6(64mn-4m-4n){\tilde{a}}^{2}{\tilde{b}}^{3}+9(28mn+2m+2n){\tilde{a}}^{3}{\tilde{b}}^{3}+12(4mn){\tilde{a}}^{3}{\tilde{b}}^{4} \end{aligned}$$

Using Table 1 we have,

$$\begin{aligned} M_2(G)= & {} 12(n+m)+4(12mn)+6(64mn-4m-4n)+9(28mn+2m+2n)+12(4mn). \\ M_2(G)= & {} 732mn+6m+6n. \end{aligned}$$

$$\square$$

### Theorem 4

Crystallographic structure of the graph of $G\approx ZnP{-}COF$.

We have:

$$\begin{aligned} ^mM_2(G)= & {} \frac{461}{54}mn+\frac{10}{9}m+\frac{10}{9}n. \end{aligned}$$

### Proof

Suppose that,

$$\begin{aligned} M(G,\tilde{a},\tilde{b})= & {} 4(n+m){\tilde{a}\tilde{b}}^{3}+(12mn){\tilde{a}}^{2}{\tilde{b}}^{2}+(64mn-4m-4n){\tilde{a}}^{2}{\tilde{b}}^{3}+(28mn+2m+2n){\tilde{a}}^{3}{\tilde{b}}^{3}+(4mn){\tilde{a}}^{3}{\tilde{b}}^{4} \end{aligned}$$

is M-Polynomial of $ZnP{-}COF$ UsinG Table 1, we have

$$\begin{aligned} f(x,{\tilde{b}})= & {} 4(n+m)x{\tilde{b}}^{3}+(12mn)x^{2}{\tilde{b}}^{2}+(64mn-4m-4n)x^{2}{\tilde{b}}^{3}+(28mn+2m+2n)x^{3}{\tilde{b}}^{3}+(4mn)x^{3}{\tilde{b}}^{4}\\ \frac{f(x,{\tilde{b}})}{x}= & {} 4(n+m){\tilde{b}}^{3}+(12mn)x{\tilde{b}}^{2}+(64mn-4m-4n)x{\tilde{b}}^{3}+(28mn+2m+2n)x^{2}{\tilde{b}}^{3}+(4mn)x^{2}{\tilde{b}}^{4} \end{aligned}$$

Apply integration on both sides.

$$\begin{aligned} \int _{0}^{{\tilde{a}}}\frac{f(x,{\tilde{b}})}{x}dx= & {} 4(n+m){\tilde{b}}^{3}\int _{0}^{{\tilde{a}}}dx+(12mn){\tilde{b}}^{2}\int _{0}^{{\tilde{a}}}xdx+(64mn-4m-4n){\tilde{b}}^{3}\int _{0}^{{\tilde{a}}}xdx\\&\qquad +&(28mn+2m+2n){\tilde{b}}^{3}\int _{0}^{{\tilde{a}}}x^{2}dx+(4mn){\tilde{b}}^{4}\int _{0}^{{\tilde{a}}}x^{2}dx\\= & {} 4(n+m){\tilde{a}\tilde{b}}^{3}+\frac{1}{2}(12mn){\tilde{a}}^{2}{\tilde{b}}^{2}+\frac{1}{2}(64mn-4m-4n){\tilde{a}}^{2}{\tilde{b}}^{3}+\frac{1}{2}(28mn+2m+2n){\tilde{a}}^{3}{\tilde{b}}^{3}+\frac{1}{3}(4mn){\tilde{a}}^{3}{\tilde{b}}^{4}\\ \end{aligned}$$

Take $$\delta _{{\tilde{b}}}$$

$$\begin{aligned} \delta _{{\tilde{b}}}= & {} \frac{1}{3}4(n+m){\tilde{a}\tilde{b}}^{3}+\frac{1}{4}(12mn){\tilde{a}}^{2}{\tilde{b}}^{2}+\frac{1}{6}(64mn-4m-4n){\tilde{a}}^{2}{\tilde{b}}^{3}+\frac{1}{6}(28mn+2m+2n){\tilde{a}}^{3}{\tilde{b}}^{3}+\frac{1}{9}(4mn){\tilde{a}}^{3}{\tilde{b}}^{4}\\ \end{aligned}$$

Now, take $$\delta _{{\tilde{a}}}$$

$$\begin{aligned} \delta _{{\tilde{a}}}\delta _{{\tilde{b}}}= & {} \frac{1}{3}4(n+m){\tilde{a}\tilde{b}}^{3}+\frac{1}{8}(12mn){\tilde{a}}^{2}{\tilde{b}}^{2}+\frac{1}{12}(64mn-4m-4n){\tilde{a}}^{2}{\tilde{b}}^{3}+\frac{1}{18}(28mn+2m+2n){\tilde{a}}^{3}{\tilde{b}}^{3}+\frac{1}{27}(4mn){\tilde{a}}^{3}{\tilde{b}}^{4}\\ \end{aligned}$$

Using Table 1, we have:

$$\begin{aligned} ^mM_2(G)= & {} \frac{1}{3}4(n+m)+\frac{1}{8}(12mn)+\frac{1}{12}(64mn-4m-4n)+\frac{1}{18}(28mn+2m+2n)+\frac{1}{27}(4mn)\\ ^mM_2(G)= & {} \frac{461}{54}mn+\frac{10}{9}m+\frac{10}{9}n. \end{aligned}$$

$$\square$$

**Table 2:** Numerical comparison of Zagreb type index polynomials.

[*m*, *n*]	[1, 1]	[2, 2]	[3, 3]	[4, 4]	[5, 5]	[6, 6]	[7, 7]	[8, 8]	[9, 9]	[10, 10]
$M_1$	580	2288	5124	9088	14180	20400	27748	36224	45828	56560
$M_2$	744	2952	6624	11760	18360	26424	35952	46944	59400	73320
$^{m}M_2$	10.75	38.59	83.50	145.48	224.53	320.66	433.87	564.14	711.50	875.92

**Figure 5:**
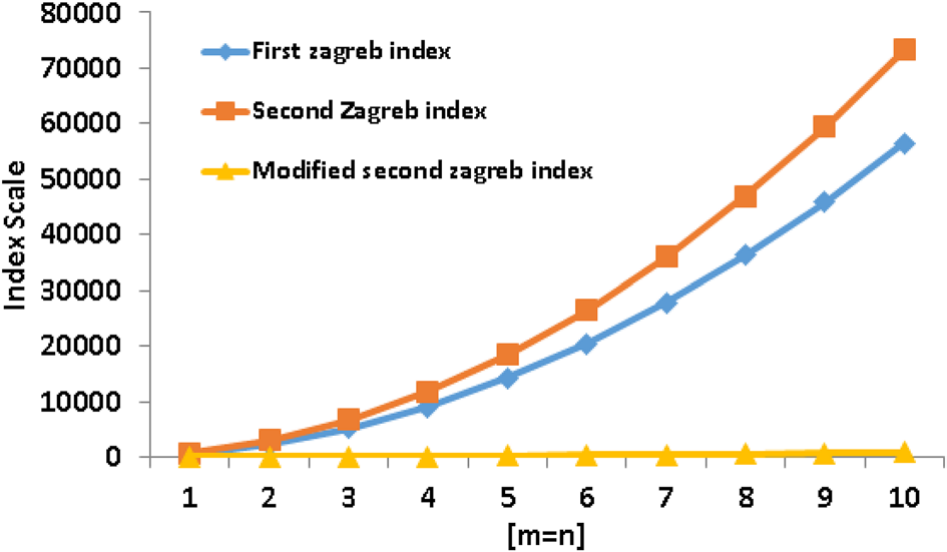
Graphical comparison of Zagreb type index polynomials.

When the second Zagreb index is compared to the other Zagreb index, an obvious trend can be seen, according to the study of Table 2 and Fig. 5. It is clear that the second Zagreb index shows a quicker increase. Its sensitivity to particular molecular graph structure elements can be attributed to this phenomenon. In computing the second Zagreb index, the squared degrees of vertices are added, highlighting higher-degree vertices and complex connection patterns. Figure 5 graphically illustrates the increased responsiveness impact on the steeper growth rate. The efficacy of the second Zagreb index in capturing complicated branching and symmetry properties inside molecular structures is demonstrated by this peculiar behavior.

### Theorem 5

Crystallographic structure of the graph of $G\approx ZnP{-}COF.$

We have:

$$\begin{aligned} R_{\alpha }(G)= & {} 3^{\alpha }4(n+m){\tilde{a}\tilde{b}}^{3}+4^{\alpha }(12mn){\tilde{a}}^{2}{\tilde{b}}^{2}+6^{\alpha }(64mn-4m-4n){\tilde{a}}^{2}{\tilde{b}}^{3}+9^{\alpha }(28mn+2m+2n){\tilde{a}}^{3}{\tilde{b}}^{3}+12^{\alpha }(4mn){\tilde{a}}^{3}{\tilde{b}}^{4}. \end{aligned}$$

### Proof

Suppose that,

$$\begin{aligned} M(G,\tilde{a},\tilde{b})= & {} 4(n+m){\tilde{a}\tilde{b}}^{3}+(12mn){\tilde{a}}^{2}{\tilde{b}}^{2}+(64mn-4m-4n){\tilde{a}}^{2}{\tilde{b}}^{3}+(28mn+2m+2n){\tilde{a}}^{3}{\tilde{b}}^{3}+(4mn){\tilde{a}}^{3}{\tilde{b}}^{4} \end{aligned}$$

is M-Polynomial of $ZnP{-}COF$ Find $D_{\tilde{a}}$

$$\begin{aligned} D_{\tilde{a}}= & {} 4(n+m){\tilde{a}\tilde{b}}^{3}+2(12mn){\tilde{a}}^{2}{\tilde{b}}^{2}+2(64mn-4m-4n){\tilde{a}}^{2}{\tilde{b}}^{3}+3(28mn+2m+2n){\tilde{a}}^{3}{\tilde{b}}^{3}+3(4mn){\tilde{a}}^{3}{\tilde{b}}^{4} \end{aligned}$$

Take $$D_{\tilde{b}}$$

$$\begin{aligned} D_{\tilde{a}}D_{\tilde{b}}= & {} 12(n+m){\tilde{a}\tilde{b}}^{3}+4(12mn){\tilde{a}}^{2}{\tilde{b}}^{2}+6(64mn-4m-4n){\tilde{a}}^{2}{\tilde{b}}^{3}+9(28mn+2m+2n){\tilde{a}}^{3}{\tilde{b}}^{3}+12(4mn){\tilde{a}}^{3}{\tilde{b}}^{4} \end{aligned}$$

Take $\alpha$ on above equation

$$\begin{aligned} D^{\alpha }_{{\tilde{a}}}D^{\alpha }_{{\tilde{b}}}= & {} 3^{\alpha }4(n+m){\tilde{a}\tilde{b}}^{3}+4^{\alpha }(12mn){\tilde{a}}^{2}{\tilde{b}}^{2}+6^{\alpha }(64mn-4m-4n){\tilde{a}}^{2}{\tilde{b}}^{3}+9^{\alpha }(28mn+2m+2n){\tilde{a}}^{3}{\tilde{b}}^{3}+12^{\alpha }(4mn){\tilde{a}}^{3}{\tilde{b}}^{4}\\ \end{aligned}$$

Using formula from Table 1, we have:

$$\begin{aligned} R_{\alpha }(G)= & {} 3^{\alpha }4(n+m){\tilde{a}\tilde{b}}^{3}+4^{\alpha }(12mn){\tilde{a}}^{2}{\tilde{b}}^{2}+6^{\alpha }(64mn-4m-4n){\tilde{a}}^{2}{\tilde{b}}^{3}+9^{\alpha }(28mn+2m+2n){\tilde{a}}^{3}{\tilde{b}}^{3}+12^{\alpha }(4mn){\tilde{a}}^{3}{\tilde{b}}^{4}\\ \end{aligned}$$

$$\square$$

### Theorem 6

Crystallographic structure of the graph of $G\approx ZnP{-}COF$

We have:

$$\begin{aligned} RR_{\alpha }(G)= & {} \frac{1}{3^\alpha }4(n+m)+\frac{1}{4^\alpha }(12mn)+\frac{1}{6^\alpha }(64mn-4m-4n)+\frac{1}{9^\alpha }(28mn+2m+2n)+\frac{1}{12^\alpha }(4mn). \end{aligned}$$

### Proof

Suppose that,

$$\begin{aligned} M(G,\tilde{a},\tilde{b})= & {} 4(n+m){\tilde{a}\tilde{b}}^{3}+(12mn){\tilde{a}}^{2}{\tilde{b}}^{2}+(64mn-4m-4n){\tilde{a}}^{2}{\tilde{b}}^{3}+(28mn+2m+2n){\tilde{a}}^{3}{\tilde{b}}^{3}+(4mn){\tilde{a}}^{3}{\tilde{b}}^{4} \end{aligned}$$

is M-Polynomial of $ZnP{-}COF$

Take $$\delta _{{\tilde{a}}}$$

$$\begin{aligned} \delta _{{\tilde{a}}}= & {} 4(n+m){\tilde{a}\tilde{b}}^{3}+\frac{1}{2}(12mn){\tilde{a}}^{2}{\tilde{b}}^{2}+\frac{1}{2}(64mn-4m-4n){\tilde{a}}^{2}{\tilde{b}}^{3}+\frac{1}{3}(28mn+2m+2n){\tilde{a}}^{3}{\tilde{b}}^{3}+\frac{1}{3}(4mn){\tilde{a}}^{3}{\tilde{b}}^{4} \end{aligned}$$

Now, take $$\delta _{{\tilde{b}}}$$

$$\begin{aligned} \delta _{{\tilde{a}}}\delta _{{\tilde{b}}}= & {} \frac{1}{3}4(n+m){\tilde{a}\tilde{b}}^{3}+\frac{1}{4}(12mn){\tilde{a}}^{2}{\tilde{b}}^{2}+\frac{1}{6}(64mn-4m-4n){\tilde{a}}^{2}{\tilde{b}}^{3}+\frac{1}{9}(28mn+2m+2n){\tilde{a}}^{3}{\tilde{b}}^{3}+\frac{1}{12}(4mn){\tilde{a}}^{3}{\tilde{b}}^{4} \end{aligned}$$

Take $\alpha$ on above equation

$$\begin{aligned} \delta {\tilde{a}}^{\alpha }_{\tilde{a}}\delta {\tilde{a}}^{\alpha }_{\tilde{b}}= & {} \frac{1}{3^\alpha }4(n+m){\tilde{a}\tilde{b}}^{3}+\frac{1}{4^\alpha }(12mn){\tilde{a}}^{2}{\tilde{b}}^{2}+\frac{1}{6^\alpha }(64mn-4m-4n){\tilde{a}}^{2}{\tilde{b}}^{3}+\frac{1}{9^\alpha }(28mn+2m+2n){\tilde{a}}^{3}{\tilde{b}}^{3}+\frac{1}{12^\alpha }(4mn){\tilde{a}}^{3}{\tilde{b}}^{4}\\ \end{aligned}$$

The Inverse Randić:

$$\begin{aligned} RR_{\alpha }(G)= & {} f({{\tilde{a}}},{\tilde{b}})|_{\tilde{a}=\tilde{b}=1} \\ RR_{\alpha }(G)= & {} \frac{1}{3^\alpha }4(n+m)+\frac{1}{4^\alpha }(12mn)+\frac{1}{6^\alpha }(64mn-4m-4n)+\frac{1}{9^\alpha }(28mn+2m+2n)+\frac{1}{12^\alpha }(4mn)\\ \end{aligned}$$

$$\square$$

**Table 3:** Numerical comparison of Randić type index polynomials.

[*m*, *n*]	[1, 1]	[2, 2]	[3, 3]	[4, 4]	[5, 5]	[6, 6]	[7, 7]	[8, 8]	[9, 9]	[10, 10]
$\alpha =1$	744	2952	6624	11760	18360	26424	35952	46944	59400	73320
$\alpha =-1$	16.14	60.25	132.58	233.14	361.92	518.92	704.14	917.58	1159.25	1429.14
$\alpha =\frac{1}{2}$	284.88	1127.02	2526.40	4483.02	6996.90	10068.02	13696.39	17882.00	22624.87	27924.98
$\alpha =-\frac{1}{2}$	38.94	148.01	328.00	578.91	900.75	1293.51	1757.19	2291.80	2897.32	3573.77

**Figure 6:**
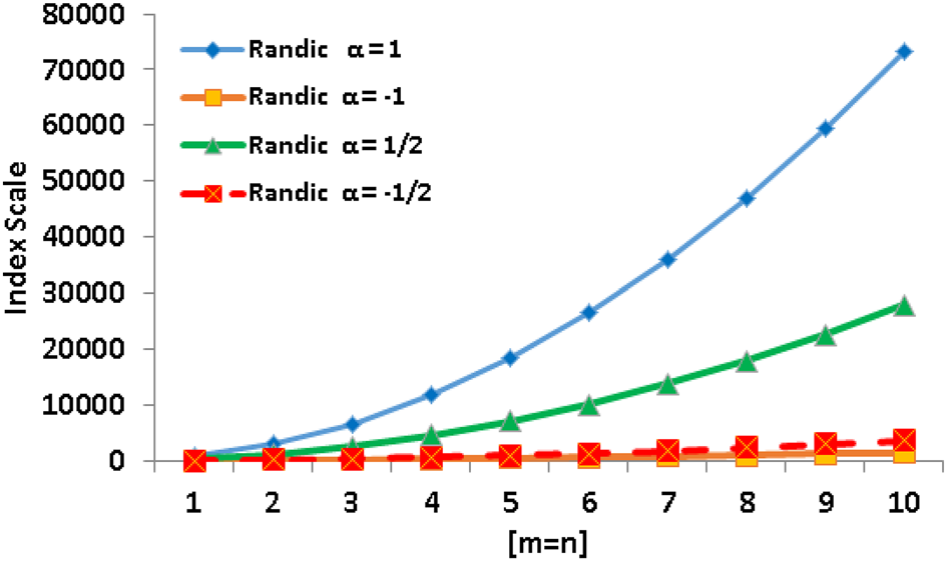
Graphical comparison of Randić index polynomials.

When comparing the behavior of the Randić index for the parameter $\alpha =1$ to the other Randić index, Table 3 and Fig. 6 reveal a clear trend. It is clear that the Randić index shows a noticeably faster growth when $\alpha =1$. These phenomena can be explained by the precise effect of the parameter alpha on the calculation of the index. When $\alpha =1$, the index gives more weight to neighboring vertex degrees, making it more sensitive to the graph’s near-immediate connection. The greater growth rate, as seen in Fig. 6, emphasizes this index increased responsiveness to regional structural configurations. This unique pattern demonstrates how well the Randić index captures and quantifies the close spatial interactions in a molecular graph. The Randić index with $\alpha =1$ is a useful tool for forecasting features that are influenced by close connection patterns as a result of this accelerated growth, underscoring its usefulness in identifying different chemical compounds with unique local structures.

### Theorem 7

Crystallographic structure of the graph of $G\approx ZnP{-}COF$.

We have:

$$\begin{aligned} SS D(G)= & {} 227mn+\frac{26}{3}m+\frac{26}{3}n. \end{aligned}$$

### Proof

Suppose that,

$$\begin{aligned} M(G,\tilde{a},\tilde{b})= & {} 4(n+m){\tilde{a}\tilde{b}}^{3}+(12mn){\tilde{a}}^{2}{\tilde{b}}^{2}+(64mn-4m-4n){\tilde{a}}^{2}{\tilde{b}}^{3}+(28mn+2m+2n){\tilde{a}}^{3}{\tilde{b}}^{3}+(4mn){\tilde{a}}^{3}{\tilde{b}}^{4} \end{aligned}$$

is M-Polynomial of $ZnP{-}COF$

Find $$\delta _{{\tilde{b}}}$$

$$\begin{aligned} \delta _{{\tilde{b}}}= & {} \frac{1}{3}4(n+m){\tilde{a}\tilde{b}}^{3}+\frac{1}{2}(12mn){\tilde{a}}^{2}{\tilde{b}}^{2}+\frac{1}{3}(64mn-4m-4n){\tilde{a}}^{2}{\tilde{b}}^{3}+\frac{1}{3}(28mn+2m+2n){\tilde{a}}^{3}{\tilde{b}}^{3}+\frac{1}{4}(4mn){\tilde{a}}^{3}{\tilde{b}}^{4} \end{aligned}$$

Now, take $$D_{\tilde{a}}$$

$$\begin{aligned} \delta _{{\tilde{b}}}D_{\tilde{a}}= & {} \frac{1}{3}4(n+m){\tilde{a}\tilde{b}}^{3}+\frac{2}{2}(12mn){\tilde{a}}^{2}{\tilde{b}}^{2}+\frac{2}{3}(64mn-4m-4n){\tilde{a}}^{2}{\tilde{b}}^{3}+\frac{3}{3}(28mn+2m+2n){\tilde{a}}^{3}{\tilde{b}}^{3}+\frac{3}{4}(4mn){\tilde{a}}^{3}{\tilde{b}}^{4} \end{aligned}$$

Find $$\delta _{{\tilde{a}}}$$

$$\begin{aligned} \delta _{{\tilde{a}}}= & {} 4(n+m){\tilde{a}\tilde{b}}^{3}+\frac{1}{2}(12mn){\tilde{a}}^{2}{\tilde{b}}^{2}+\frac{1}{2}(64mn-4m-4n){\tilde{a}}^{2}{\tilde{b}}^{3}+\frac{1}{3}(28mn+2m+2n){\tilde{a}}^{3}{\tilde{b}}^{3}+\frac{1}{3}(4mn){\tilde{a}}^{3}{\tilde{b}}^{4} \end{aligned}$$

Now, take $$D_{\tilde{b}}$$

$$\begin{aligned} \delta _{{\tilde{a}}}D_{\tilde{b}}= & {} 34(n+m){\tilde{a}\tilde{b}}^{3}+\frac{2}{2}(12mn){\tilde{a}}^{2}{\tilde{b}}^{2}+\frac{3}{2}(64mn-4m-4n){\tilde{a}}^{2}{\tilde{b}}^{3}+\frac{3}{3}(28mn+2m+2n){\tilde{a}}^{3}{\tilde{b}}^{3}+\frac{4}{3}(4mn){\tilde{a}}^{3}{\tilde{b}}^{4} \end{aligned}$$

Using Table 1 we have,

$$\begin{aligned} SS D(G)= & {} \left[ \frac{1}{3}4(n+m)+\frac{2}{2}(12mn)+\frac{2}{3}(64mn-4m-4n)+\frac{3}{3}(28mn+2m+2n)+\frac{3}{4}(4mn)\right] \\&\qquad +&\left[ 34(n+m)+\frac{2}{2}(12mn)+\frac{3}{2}(64mn-4m-4n)+\frac{3}{3}(28mn+2m+2n)+\frac{4}{3}(4mn)\right] \\ SS D(G)= & {} 227mn+\frac{26}{3}m+\frac{26}{3}n. \end{aligned}$$

$$\square$$

### Theorem 8

Crystallographic structure of the graph of $G\approx ZnP{-}COF[m; n]$, where $n; m\ge 1$

We have:

$$\begin{aligned} H(G)= & {} \frac{2209}{105}mn+\frac{8}{15}m+\frac{8}{15}n. \end{aligned}$$

### Proof

Suppose that,

$$\begin{aligned} M(G,\tilde{a},\tilde{b})= & {} 4(n+m){\tilde{a}\tilde{b}}^{3}+(12mn){\tilde{a}}^{2}{\tilde{b}}^{2}+(64mn-4m-4n){\tilde{a}}^{2}{\tilde{b}}^{3}+(28mn+2m+2n){\tilde{a}}^{3}{\tilde{b}}^{3}+(4mn){\tilde{a}}^{3}{\tilde{b}}^{4} \end{aligned}$$

is M-Polynomial of $ZnP{-}COF$

Find $$Jf({{\tilde{a}}},{\tilde{b}})$$

$$\begin{aligned} Jf({{\tilde{a}}},{\tilde{b}})=Jf(a,a)= & {} 4(n+m)a{\tilde{a}}^{3}+(12mn){\tilde{a}}^{2}{\tilde{a}}^{2}+(64mn-4m-4n){\tilde{a}}^{2}{\tilde{a}}^{3}+(28mn+2m+2n){\tilde{a}}^{3}{\tilde{a}}^{3}+(4mn){\tilde{a}}^{3}{\tilde{a}}^{4}\\= & {} 4(n+m){\tilde{a}}^{4}+(12mn){\tilde{a}}^{4}+(64mn-4m-4n){\tilde{a}}^{5}+(28mn+2m+2n){\tilde{a}}^{6}+(4mn){\tilde{a}}^{7} \end{aligned}$$

Take $$\delta _{{\tilde{a}}}$$

$$\begin{aligned} \delta _{{\tilde{a}}}Jf({{\tilde{a}}},{\tilde{b}})= & {} \frac{1}{4}4(n+m){\tilde{a}}^{4}+\frac{1}{4}(12mn){\tilde{a}}^{4}+\frac{1}{5}(64mn-4m-4n){\tilde{a}}^{5}+\frac{1}{6}(28mn+2m+2n){\tilde{a}}^{6}+\frac{1}{7}(4mn){\tilde{a}}^{7} \end{aligned}$$

The Harmonic index:

$$\begin{aligned} H(G)= & {} 2(\delta _{{\tilde{a}}}Jf({{\tilde{a}}},{\tilde{b}}))|_{a=1} \\ H(G)= & {} \frac{1}{4}4(n+m)+\frac{1}{4}(12mn)+\frac{1}{5}(64mn-4m-4n)+\frac{1}{6}(28mn+2m+2n)+\frac{1}{7}(4mn) \\ H(G)= & {} \frac{2209}{105}mn+\frac{8}{15}m+\frac{8}{15}n. \end{aligned}$$

$$\square$$

### Theorem 9

Crystallographic structure of the graph of $G\approx ZnP{-}COF$. We have:

$$\begin{aligned} \delta _{{\tilde{a}}}JD_{\tilde{b}}D_{\tilde{a}}= & {} \frac{4818}{35}mn+\frac{6}{5}m+\frac{6}{5}n. \end{aligned}$$

### Proof

Suppose that,

$$\begin{aligned} M(G,\tilde{a},\tilde{b})= & {} 4(n+m){\tilde{a}\tilde{b}}^{3}+(12mn){\tilde{a}}^{2}{\tilde{b}}^{2}+(64mn-4m-4n){\tilde{a}}^{2}{\tilde{b}}^{3}+(28mn+2m+2n){\tilde{a}}^{3}{\tilde{b}}^{3}+(4mn){\tilde{a}}^{3}{\tilde{b}}^{4} \end{aligned}$$

is M-Polynomial of $ZnP{-}COF$

Take $$D_{\tilde{b}}$$

$$\begin{aligned} D_{\tilde{b}}= & {} 34(n+m){\tilde{a}\tilde{b}}^{3}+2(12mn){\tilde{a}}^{2}{\tilde{b}}^{2}+3(64mn-4m-4n){\tilde{a}}^{2}{\tilde{b}}^{3}+3(28mn+2m+2n){\tilde{a}}^{3}{\tilde{b}}^{3}+4(4mn){\tilde{a}}^{3}{\tilde{b}}^{4} \end{aligned}$$

Now, take $$D_{\tilde{a}}$$

$$\begin{aligned} D_{\tilde{b}}D_{\tilde{a}}= & {} 34(n+m){\tilde{a}\tilde{b}}^{3}+4(12mn){\tilde{a}}^{2}{\tilde{b}}^{2}+6(64mn-4m-4n){\tilde{a}}^{2}{\tilde{b}}^{3}+9(28mn+2m+2n){\tilde{a}}^{3}{\tilde{b}}^{3}+12(4mn){\tilde{a}}^{3}{\tilde{b}}^{4} \end{aligned}$$

Find $$JD_{\tilde{b}}D_{\tilde{a}}$$

$$\begin{aligned} JD_{\tilde{b}}D_{\tilde{a}}= & {} 34(n+m)a{\tilde{a}}^{3}+4(12mn){\tilde{a}}^{2}{\tilde{a}}^{2}+6(64mn-4m-4n){\tilde{a}}^{2}{\tilde{a}}^{3}+9(28mn+2m+2n){\tilde{a}}^{3}{\tilde{a}}^{3}+12(4mn){\tilde{a}}^{3}{\tilde{a}}^{4}\\= & {} 34(n+m){\tilde{a}}^{4}+4(12mn){\tilde{a}}^{4}+6(64mn-4m-4n){\tilde{a}}^{5}+9(28mn+2m+2n){\tilde{a}}^{6}+12(4mn){\tilde{a}}^{7} \end{aligned}$$

Take $$\delta _{{\tilde{a}}}$$

$$\begin{aligned} \delta _{{\tilde{a}}}JD_{\tilde{b}}D_{\tilde{a}}= & {} \frac{3}{4}4(n+m){\tilde{a}}^{4}+(12mn){\tilde{a}}^{4}+\frac{6}{5}(64mn-4m-4n){\tilde{a}}^{5}+\frac{9}{6}(28mn+2m+2n){\tilde{a}}^{6}+\frac{12}{7}(4mn){\tilde{a}}^{7} \end{aligned}$$

The Inverse sum index:

$$\begin{aligned} \delta _{{\tilde{a}}}JD_{\tilde{b}}D_{\tilde{a}}|_{a=1}= & {} \frac{3}{4}4(n+m)+(12mn)+\frac{6}{5}(64mn-4m-4n)+\frac{9}{6}(28mn+2m+2n)+\frac{12}{7}(4mn) \\ \delta _{{\tilde{a}}}JD_{\tilde{b}}D_{\tilde{a}}= & {} \frac{4818}{35}mn+\frac{6}{5}m+\frac{6}{5}n. \end{aligned}$$

$$\square$$

**Table 4:** Numerical comparison of different index polynomials.

[*m*, *n*]	[1, 1]	[2, 2]	[3, 3]	[4, 4]	[5, 5]	[6, 6]	[7, 7]	[8, 8]	[9, 9]	[10, 10]
*SSD*(*G*)	244.33	942.67	2095.00	3701.33	5761.67	8276.00	11244.33	14666.67	18543.00	22873.33
*H*(*G*)	22.10	86.29	192.54	340.88	531.29	763.77	1038.33	1354.97	1713.69	2114.48
$\delta _{{\tilde{a}}}JD_{\tilde{b}}D_{\tilde{a}}$	140.06	555.43	1246.11	2212.11	3453.43	4970.06	6762.00	8829.26	11171.83	13789.71

**Figure 7:**
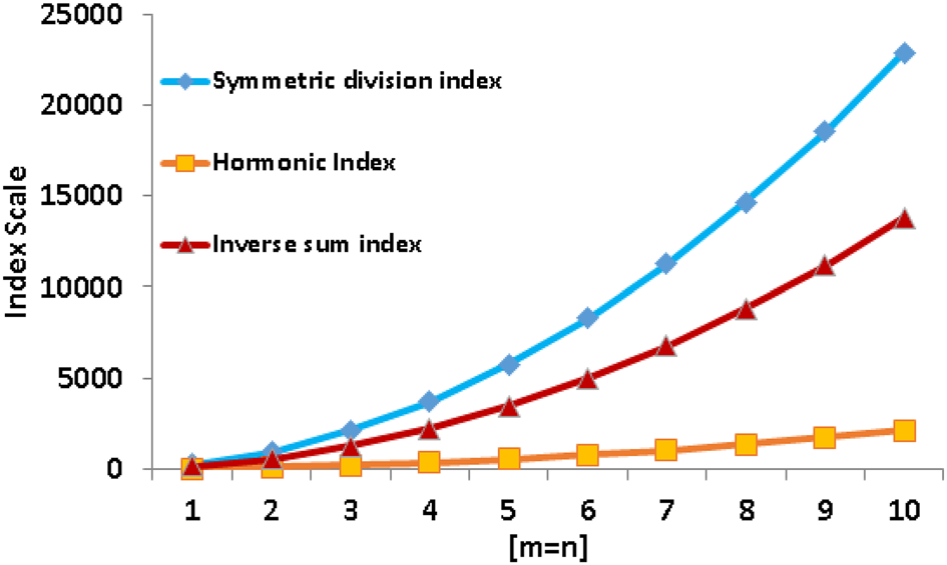
Graphical comparison of different index polynomials.

The comparison of the data in Table 4 and the graphical display in Fig. 7 reveals a clear pattern regarding the rate of increase for various indices. Particularly, as compared to both the harmonic index and the inverse sum index, the symmetric division index shows a substantially faster rise. The symmetric division index’s distinctive mathematical properties, which more clearly highlight changes in graph structure, are credited with this insight. The index’s enhanced sensitivity to minute structural details inside molecular graphs is highlighted by this rapid trend. The relevance of the symmetric division index in capturing subtle molecular subtleties is highlighted by the graphically appealing representation in Fig. 7. As a result, it appears that the symmetric division index has a clear advantage over the harmonic and inverse sum indices for identifying the intricate molecular architecture, allowing it to offer insightful information for some structural investigations and prognostication.

## Conclusion

The degree-based topological indices of the two-dimensional ZnP-COF were explored in this study, including noteworthy indices. Furthermore, topological indices for the two-dimensional ZnP-COF were derived using a variety of common polynomials. The knowledge gained from this work has the potential to be applied in the fields of chemical and pharmaceutical research by being incorporated into several Quantitative Structure-Activity Relationship (QSAR) models. By calculating additional topological indices for the two-dimensional ZnP-COF based on degrees and counts, this study expands the field and advances our understanding of the structural characteristics of this material.

## Data Availability

The datasets used and/or analysed during the current study available from the corresponding author on reasonable request.
